# Differential expression of miRNAs in bronchoalveolar lavage fluid and plasma from patients with chronic obstructive pulmonary disease

**DOI:** 10.1097/MD.0000000000030969

**Published:** 2022-10-07

**Authors:** Jianwu Hu, Weina Wang, Qiaofa Lu, Lifen Du, Tian Qin

**Affiliations:** a Department of Pulmonary and Critical Care Medicine, Wuhan Fourth Hospital, Wuhan, Hubei, China; b Department of Pharmacy, Wuhan Fourth Hospital, Wuhan, Hubei, China.

**Keywords:** bioinformatic analysis, chronic obstructive pulmonary disease, differential expression, miRNAs, second-generation sequencing

## Abstract

Micro RNAs (MiRNAs) act as a key regulator participating in various biological process, and the roles of that play in chronic obstructive pulmonary disease (COPD) are discovered. However, recent pharmacological treatment for COPD focus on alleviating symptoms and reducing the risk events. The heterogeneous COPD causes variable responses to pharmacological interventions. COPD treatment has gradually developed into precision medicine, integrating clinical and biomarker information to optimize personalized therapy. Thus, targeting miRNAs represents a promising strategy for COPD individual therapy. Twelve COPD patients, 7 community-acquired pneumonia and 4 normal people were recruited. Total RNAs were collected from the bronch alveolar lavage cells and peripheral blood plasma of each participant. miRNAs were profiled by microarray and systematically compared between patients with different groups. Bioinformatic analysis identified pathways relevant to the pathogenesis of COPD. Next, the target pathway networks were mapped. Compared different groups, we obtain differential expression of miRNAs (*Q* value (Adjusted *P* value) < .05 and |log^2^FC| >2). Gene ontology enrichment analyses showed that differentially expressed miRNAs function as regulators in different modules of cellular component, molecular function and biological process. Kyoto Encyclopedia of Genes and Genomes enrichment analyses suggested that signals, such as MAPK signaling pathway, Ras signaling pathway, FoxO signaling pathway and oxidative stress may participate in the pathogenesis of COPD. In the miRNAs target pathway networks, novel-hsa-miR26-3p or hsa-miR-3529-3p/CDC42/MAPK signaling pathway may play a role in regulating COPD. Our findings demonstrate critical roles of the miRNAs in COPD molecular pathology. The data support a plausible mechanism that miRNAs may be involved in the development of COPD by affecting the inflammatory and oxidative stress. Moreover, hsa-miR-4748/CDC42/MAPK signaling pathway may contribute to the pathogenesis of COPD, providing a potential novel therapeutic strategy in COPD.

## 1. Introduction

Chronic obstructive pulmonary disease (COPD) is characterized by incompletely reversible airway obstruction. The statistical data from World health organization show that its morbidity and mortality rates are still on the rise, and it is estimated to be the third leading cause of death in the world by 2030.^[1]^ Previous studies on COPD pathogenesis are mainly concentrated in imbalance between proteolysis and antiproteolysis, inflammatory cell infiltration in the lung, oxidative stress and apoptotic disorders, cellular senescence, etc.^[2–5]^ With the development of genomics and proteomics, the biological effects of micro RNA (miRNAs) are gradually understood in the COPD pathophysiology.

MiRNAs are a class of endogenous and noncoding single-stranded ribonucleic acid (RNA) at length of 19 to 25 nucleotides, through combining the 3’-end nontranslation zone of the purpose mRNAs to inhibit mRNAs translation or promote its complete degradation, acting as a key regulator participating in cell proliferation, differentiation, development and apoptosis.^[6–8]^ The roles of miRNAs play in COPD are gradually discovered. However, recent pharmacological treatment for COPD focus on alleviating symptoms and reducing the risk events.^[9]^ The heterogeneous COPD causes variable responses to pharmacological interventions.^[10]^ COPD treatment has gradually developed into precision medicine, integrating clinical and biomarker information to optimize personalized therapy.^[11–13]^ Thus, targeting miRNAs represents a promising strategy for COPD individual therapy.

In our study, we applied second-generation sequencing method to explore differential expression profiles of miRNAs in bronchoalveolar lavage fluid (BALF) and plasma from patients with COPD and conduct bioinformatic analysis, to providing a potential novel therapeutic strategy in COPD.

## 2. Methods

### 2.1. Subjects selection

Twelve COPD patients hospitalized from November 2020 to April 2021 in Wuhan Fourth Hospital were included in the treatment group (Subjects were classified in the COPD group if they had a post bronchodilator forced vital capacity rate of 1 second of less than 0.70). Seven community-acquired pneumonia (CAP) patients hospitalized at the same period and 4 health people were included in control group, whose lung function were normal. This study was approved by the ethics committee of medical research in Wuhan Fourth Hospital (No. KY2020-137-01). Written informed consent was obtained from participants prior to their enrollment in the study. The demographic characteristics of each group were collected. According to the requirement of repeated analysis and biology, the 3 samples of BALF and 9 samples of plasma of each group were collected. Three samples of BALF from COPD and no-COPD patients were collected, named BALF group and BALF-control group. Nine samples of peripheral blood plasma were collected, named COPD group and control group.

This study needed to collect BALF cells, which is an invasive operation with certain risks and pains. For medical ethics, people with CAP was selected as the research object to collect BALF cells.

### 2.2. Sample collection and miRNA extraction

For the samples from BALF, the patient was locally anesthetized with 1% lidocaine injection at oropharynx, and administrated 5 mg of midazolam intravenously. Then, patients were maintained at supine position and Implemented oxygen, ECG, blood pressure monitoring. Apply fiber bronchoscope to conduct broncholaveolar lavage. A total of 60 mL of physiological saline heating to 37°C was infused, recovered and placed in 15 mL RNase-free tube, stored at 4°C and centrifuged at 8000 *g* for10 minutes. The supernatant was discard. The cell pellet was transferred to a 1.5 mL RNase-free eppendorf tube and frozened at −80°C. RNA was extracted within 1 week.

For the samples from peripheral blood plasma 2 mL of venous blood was drawed from elbow vein and placed in ethylene diamine tetraacetic acid tube. The sample were centrifuged at 3000 *g* for 8 minutes. The upper plasma was transfered to 1.5 mL RNase-free eppendorf tube and frozened at −80°C. RNA was extracted within 1 week.

The miRNA was extracted by BGI Biotechnology Corporation (Wuhan, China) in accordance with standard miRNA extraction methods. The total RNA was qualified and quantified using a Nano Drop and Agilent 2100 bioanalyzer (Thermo Fisher Scientific, MA, USA).

### 2.3. MiRNA library construction and microarray

The miRNAs that met the sequencing criteria were further included in the library and sequenced by MGISEQ2000 platform (MGI-Shenzhen, China) and differentially expressed genes were obtained by DEseq2 method. Hierarchical clustering analysis of differential miRNAs was performed using heatmap function in R software (R Foundation for Statistical Computing, Vienna, Austria). The target gene prediction was conducted by comparing the reference gene with target gene prediction software (RNAhybrid, miRanda and TargetScan).

The expression profiles were assessed by microarray according to the recommended protocols provided by BGI Biotechnology Corporation (Wuhan, China).

### 2.4. Functional analysis and miRNA target pathway network

Gene Ontology (GO) analyses and subsequent Kyoto Encyclopedia of Genes and Genomes (KEGG) molecular pathway enrichment analyses were performed based on the predicted results of target genes. GO analysis was conducted by GO: TermFinder software (Stanford University, Stanford, CA) (http://www.yeastgenome.org/help/analyze/go-term-finder).

The target genes of miRNA were predicted based on miRanda software (V3.3a; www.microrna.org) and Targetscan (v7.2; www.targetscan.org). And then top5 mRNAs for each miRNA (sorted by free energy) were selected and Cytoscape software (V3.7.1, the U.S. National Institute of General Medical Sciences, Maryland) was used to map the network.

### 2.5. Statistical analysis

Statistical analyses were conducted with the Statistical Package for Social Sciences software package (SPSS, version 22.0 [IBM Corporation, Armonk, NY]). The patients indicators comply with the normal distribution. The average ± standard deviation is used to describe the variance. Then the homogeneity test of variances is used. If equal variance, *t* test is applied; if not, adjusted *t* test is applied. An adjusted *Q* value (Adjusted *P* value) < .05 and |log^2^FC| >2 were set as the threshold for identifying differentially expressed miRNAs. Other analyses were set at *Q* value < .05.

## 3. Results

### 3.1. The clinical characteristics of subjects

A total of 12 COPD patients, 7 CAP patients and 4 normal people were included in our study. The general information of 2 groups was showed in Table 1.

**Table 1:** Characteristics and respiratory parameters of COPD patients and controls.

	COPD group (n = 12)	Control group (n = 11)	*P*
Age	60.75 ± 13.00	51.82 ± 21.58	.251
FVC (% pred)	80.00 ± 27.46	91.64 ± 20.91	.269
FEV_1_/FVC, %	43.00 ± 12.02	79.09 ± 5.43	.001

COPD = chronic obstructive pulmonary disease, FVC = forced vital capacity, FVC% pred = percentage of FVC in the predicted value, FEV_1_/FVC = forced vital capacity rate of 1 second.

### 3.2. The results of sequencing

A total of 24 samples were sequenced on DNBSEQ platform, with an average yield of 33.87 M reads per sample. This BioProject accession number is PRJNA847376. The average alignment ratio of the sample comparison genome was 78.22%. A total of 1,215 miRNAs were detected, including known-miRNAs 989 and novel-miRNAs 226, whereas 13 did not have expressions.

### 3.3. MiRNA microarray

**Figure 1.**
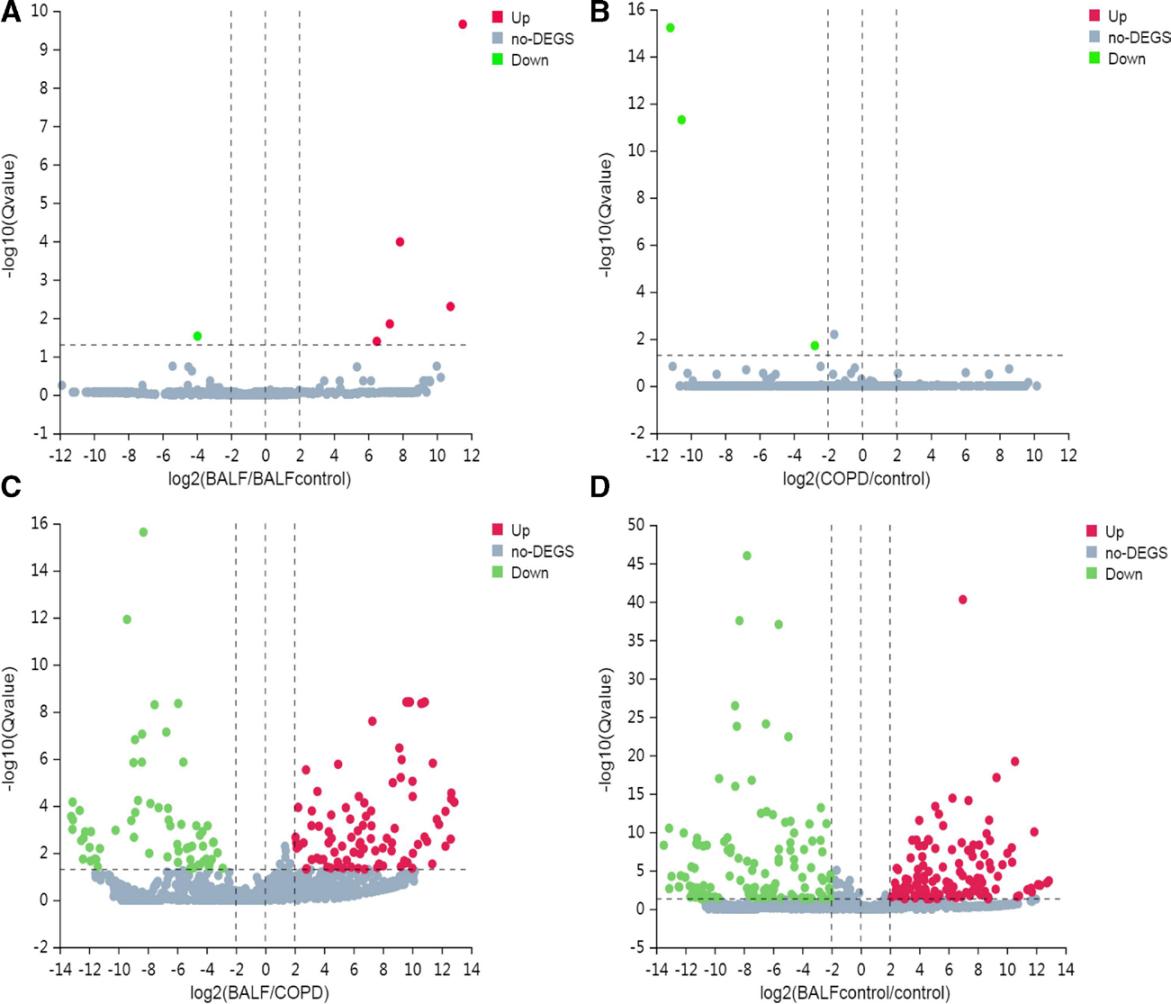
MiRNA expression profile of different group. Volcano plots were used to distinguish DEmiRNAs. Red, green and gray indicate up-regulation, down-regulation and no DEGS, respectively. DEGS = differential expressed genes, DEmiRNAs = differential expressed miRNAs, MiRNAs = micro RNAs.

Then, screened miRNAs were conducted by microarray. The parameter was set as *Q* value (Adjusted *P* value) < .05 and |log^2^FC| >2. The results showed that, compared with BALF control group, there was 5 up-regulated miRNAs and 1 up-regulated miRNA in BALF group (Table 2). Compared with control group, there was 3 down-regulated miRNAs in COPD group, and no miRNAs upregulation (Table 2). We also compared differential expressed miRNAs (DEmiRNAs) in BALF and plasma of the COPD patients, and obtained 93 up-regulated miRNAs and 66 down-regulated miRNAs, among which there was 7 novel up-regulated and 6 novel down-regulated miRNAs, the top 10 of which was listed in Table 2. The Volcano plots of DEmiRNAs in 4 groups were made (Fig. 1A–D).

**Table 2:** DEmiRNAs between BALF and BALFcontrol, COPD group and control, and the top ten DEmiRNAs between BALF group and COPD group.

Gene ID	log^2^ (BALF/BALFcontrol)	*Q* value (BALF/BALFcontrol)
hsa-miR-129-5p	6.502317431	0.040359584
hsa-miR-3529-3p	7.857561109	1.04E-04
hsa-miR-365b-3p	11.5137276	2.23E-10
hsa-miR-6503-5p	10.80251637	0.004993365
novel-hsa-miR26-3p	7.260079439	0.014240805
hsa-miR-34b-5p	−3.95506095	0.029557731
Gene ID	log^2^ (COPD/control)	*Q* value (COPD/control)
hsa-miR-4748	−10.53527538	4.91E-12
hsa-miR-491-5p	−2.764433491	0.019400655
novel-hsa-miR158-3p	−11.19414124	6.00E-16
Gene ID	log^2^(BALF/ COPD)	*Q* value (BALF/ COPD)
hsa-miR-1227-3p	−13.21112782	2.80E-04
hsa-miR-4435	−13.12686577	9.90E-04
novel-hsa-miR220-3p	−13.11618149	6.90E-05
novel-hsa-miR254-3p	−13.09308739	3.92E-04
hsa-miR-1260b	−12.6329952	1.57E-04
hsa-miR-6749-3p	−12.52747701	0.002990671
hsa-miR-2115-5p	12.61332905	0.002581809
hsa-miR-449c-5p	12.64205169	5.04E-05
novel-hsa-miR54-5p	12.65575431	2.85E-05
hsa-miR-7704	12.86069903	6.98E-05

BALF = bronchoalveolar lavage fluid, COPD = chronic obstructive pulmonary disease, DEmiRNAs = differential expressed miRNAs.

### 3.4. The differences in miRNAs expression levels

We also detected miRNAs expression quantity between groups. Compared with control group, there is 141 DEmiRNAs in BALF group and 124 DEmiRNAs in COPD group. There was 12 miRNAs expressing both in the COPD group and the BALF instead of 2 control groups, they are hsa-miR-2276-3p, hsa-miR-550a-3-5p, hsa-miR-616-5p, hsa-miR-5684, hsa-miR-6501-3p, hsa-miR-4795-5p, hsa-miR-320e, hsa-miR-1268b, hsa-miR-301b-3p, hsa-miR-4634, hsa-miR-136-5p and hsa-miR-4787-3p.

### 3.5. The cluster heatmaps of DEmiRNAs

**Figure 2.**
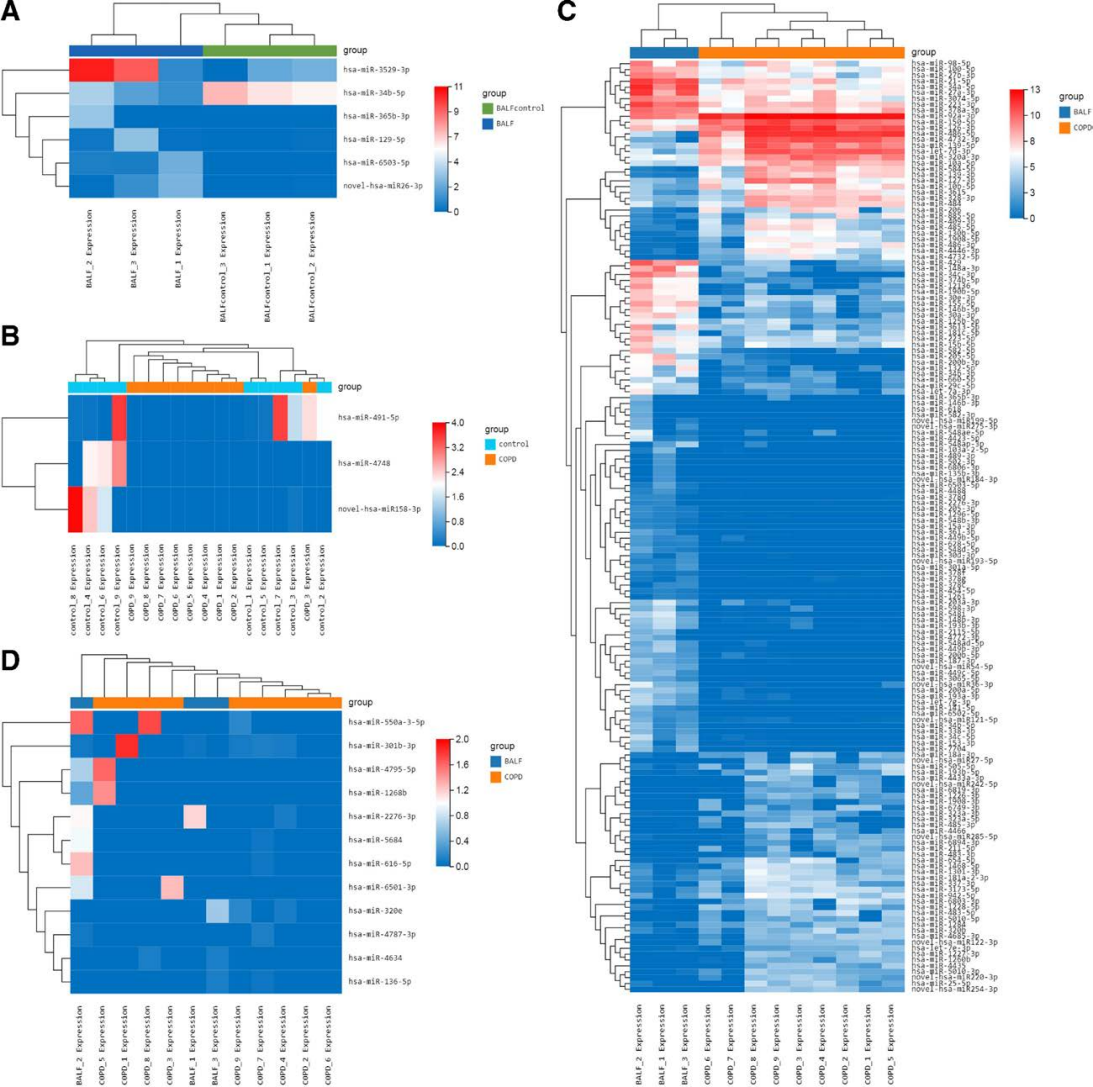
The cluster heatmaps of DEmiRNAs from different group. (A–C) The cluster heatmaps of BALF control-vs-BALF group, control-vs-COPD group and COPD-vs-BALF group. (D) The cluster heatmaps of 12 DEmiRNAs both expressed in BALF and plasma. BALF = bronchoalveolar lavage fluid, COPD = chronic obstructive pulmonary disease, DEmiRNAs = differential expressed miRNAs, MiRNAs = micro RNAs.

The cluster heatmaps of 6 DEmiRNAs in BALF group and BALF control group was showed in Figure 2A, and that of 3 DEmiRNAs in COPD group and control group was showed in Figure 2B, and that of 159 DEmiRNAs in COPD group and BALF group was showed in Figure 2C. There is no difference between COPD group and control group. As showed in Figure 2D, there is 12 DEmiRNAs expressing only in BALF and plasma from patients. The color box displays the levels of DEmiRNAs.

### 3.6. GO enrichment analyses

The GO enrichment analyses were performed in DEmiRNAs from BALFcontrol-vs-BALF group, control-vs-COPD group and COPD-vs-BALF group, which consisting cellular component (C), molecular function (F) and biological process (P). The 6 DEmiRNAs in BALF group mainly enriched in the function of cell junction, kinase activity, etc. The 3 DEmiRNAs in plasma from COPD were concentrated on adherens junction, Wnt signaling pathway, etc. The 159 DEmiRNAs were mainly focused on cell adhesion, intracellular signal transduction, etc. The 12 DEmiRNAs both expressed in BALF and COPD group were discovered in intracellular signal transduction, cell adhesion, etc.

### 3.7. KEGG analyses of target genes

**Figure 3.**
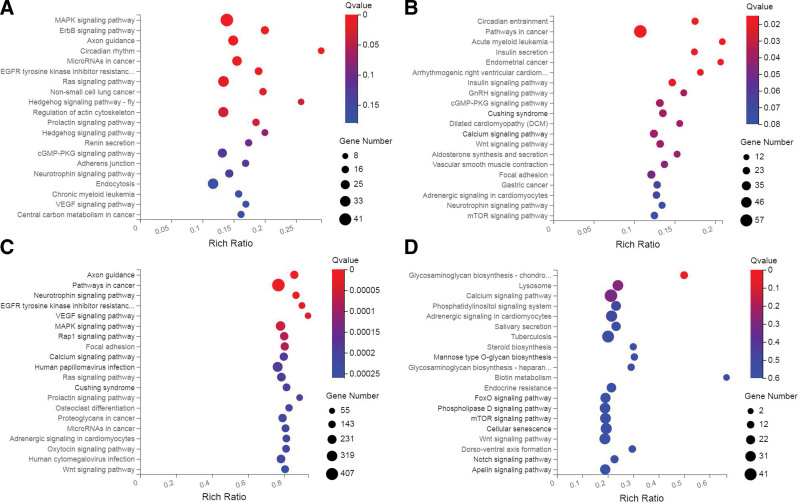
KEGG analyses of target genes of different group. (A–C) KEGG analyses in BALF control-vs-BALF group, control-vs-COPD group and COPD-vs-BALF group. (D) KEGG analyses of 12 DEmiRNAs both expressed in BALF and COPD group instead in 2 control group. BALF = bronchoalveolar lavage fluid, COPD = chronic obstructive pulmonary disease, DEmiRNAs = differential expressed miRNAs, KEGG = Kyoto encyclopedia of genes and genomes, MiRNAs = micro RNAs.

KEGG analyses were conducted in BALFcontrol-vs-BALF group, control-vs-COPD group and COPD-vs-BALF group (Fig. 3A–C). The 6 DEmiRNAs in BALF group were predicted to regulate 1,405 mRNA, 309 pathways were enriched by KEGG, significant enrichment top 20 pathways was showed in Figure 3A. Among them, the pathways related to COPD was MAPK signaling pathway, ErbB signaling pathway, Ras signaling pathway, etc. The 3 DEmiRNAs in COPD group were predicted to regulate 1,308 mRNA, 299 pathways were enriched by KEGG, significant enrichment top 20 pathways was showed in Figure 3B. Among them, the pathways related to COPD was Pathways in cancer, Wnt signaling pathway, Focal adhesion, etc. Compared COPD group and BALF group, the 159 DEmiRNAs in were predicted to regulate 12,474 mRNA, 343 pathways were enriched by KEGG, significant enrichment, top 20 pathways was showed in Figure 3C. Among them, the pathways related to COPD was VEGF signaling pathway, MAPK signaling pathway, Ras signaling pathway, Oxidative phosphorylation, etc. The 12 DEmiRNAs both expressed in BALF and COPD group instead in 2 control group were analyzed and discovered in FoxO signaling pathway, Wnt signaling pathway, Ras signaling pathway, etc (Fig. 3D).

### 3.8. Target prediction and network analysis.

**Figure 4.**
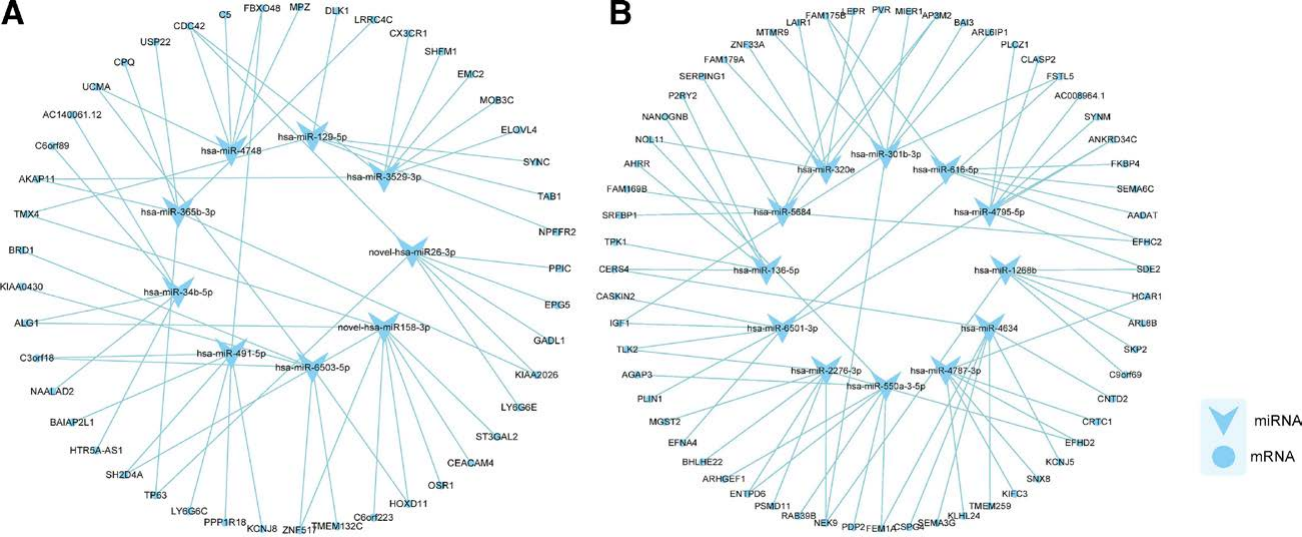
The networks of miRNA. (A) The network constructed on 6 DEmiRNAs relatively down-regulated in BALF group and 3 DEmiRNAs relatively down-regulated in COPD group. (B) The network constructed on the 12 DEmiRNAs expressed both in BALF and plasma from patients. BALF = bronchoalveolar lavage fluid, COPD = chronic obstructive pulmonary disease, DEmiRNAs = differential expressed miRNAs, MiRNAs = micro RNAs.

To predict the possible functions of the DEmiRNAs, the miRNA-RNA target pathway networks were mapped. We chose the 6 DEmiRNAs relatively down-regulated in BALF group and 3 DEmiRNAs relatively down-regulated in COPD group to construct the network. As showed in Figure 4A, novel-hsa-miR26-3p or hsa-miR-3529-3p/CDC42/MAPK signaling pathway may play a role in the regulation of COPD. In addition, hsa-miR-129-5p were predicted to target TAB1, which was in connection to MAPK signaling pathway. The 12 DEmiRNAs expressed both in BALF and plasma from patients was chosen to map the network, and found that hsa-miR-1268b was forecast to target SKP2, which was related to FoxO signaling pathway and hsa-miR-6501-3p was predicted to target EFNA4, which was also associated with MAPK signaling pathway (Fig. 4B).

## 4. Discussion

The diagnosis of COPD currently mainly relies on clinical symptoms and pulmonary function tests. However, patients with mild and moderate COPD usually lack the typical symptoms, resulting that they do not receive effective attention and miss the opportunity for early diagnosis and timely intervention. In recent decades, with the development of genomics and proteomics, miRNAs have been found to be involved in the pathological development of many lung diseases such as COPD, lung cancer, pulmonary fibrosis, bronchitis, asthma, etc. The miRNAs present in serum and plasma, namely circulating miRNAs, may become early diagnostic markers for various diseases.

It has been reported that some differentially expressed miRNAs were obtained from sputum, plasma and lung tissue of COPD patients. Van Pottelberge GR et al applied reverse transcription-PCR to detect the differences in the expression of 627 known miRNAs in the induced sputum of smoking patients with COPD and nonsmoking normal subjects and found that the expression levels of let-7c and miR-125b were significantly down-regulated in COPD group, and the expression of let-7c target gene TNFR-II and its protein also showed corresponding changes.^[14]^ Akbas F et al detected 72 miRNAs in peripheral serum of COPD patients by quantitative reverse transcription PCR array, and found that the expression levels of miR-20a, miR-28-3p, miR-34c-5p, and miR-100 were down-regulated, while the expression level of miR-7 was up-regulated.^[15]^ Cao et al detected increased expression of miR-183, miR-200b and miR-200c in the peripheral blood of COPD patients.^[16]^ Kara et al noticed miR-29c and miR-126 expressions showed significant differences in stage III and only miR-126 different in stage IV in peripheral blood.^[17]^ Lacedonia D et al discovered miRNA-338 is higher in the supernatant than in peripheral blood.^[18]^ Ezzie ME et al found that miR-15b, miR-223, miR-1274a, and miR-424 were significantly up-regulated in lung tissue of COPD patients and were associated with inflammatory pathways or signaling molecules such as TGF-*β*, Wnt, and SMAD7.^[19]^ Gu et alreported miR-195 was significantly up-regulated in the lung tissues and regulates Akt signaling by suppressing PH domain and leucine rich repeat protein phosphatases 2 expression.^[20]^ Shi et al found that the expression of miR-181a, miR-203, miR-338, miR-1 and miR-199a was altered in the lung biopsies and only miR-203 was higher in the blood.^[21]^ Molina-Pinelo S et al found that around 50% miRNAs were no detected in both plasma and bronchoalveolar cell fraction and only 20% of miRNAs showed similar expression in both samples, like miR-17, miR-19b, miR-195 and miR-20b.^[22]^

These studies rarely reach consistent conclusions, some of which may be contributed to different detection methods, and the other part is also related to different sample sources. Given that the differences between animal models or cell experiments and the real and complex pathophysiological processes of the human body, the suitable sample to obtain for studying the pathogenesis of COPD is cells from the BALF. However, such samples also have a small number of cells and a low amount of miRNA extraction, which are precious samples, and the detection by RT-PCR and miRNA is incomplete. In our study, next-generation sequencing was applied to detect differentially expressed miRNAs in BALF samples, making sure that the detection of miRNAs in precious samples was more comprehensive. The plasma samples from patients were selected for comparative analysis to clarify the differences in the expression of miRNAs in BALF and plasma, and it is expected to discover the specificity of expression in diseased tissues.

In our experiments, differentially expressed miRNAs from BALF and plasma were obtained. It has been reported that these miRNAs play a role in angiogenesis, metastasis and recurrence of various tumors. For instance, the miR-34b family is a well-known cancer suppressor.^[23]^ hsa-miR-129-5p is down-regulated in lung cancer tissues.^[24]^ The down-regulation of PART1 can hinder the occurrence and development of liver cancer by targeting the hsa-miR-3529-3p/FOXC2 axis.^[25]^ The hsa-miR-365b-3p has been identified as a novel miRNA described in various types of tumor including non-small cell lung cancer^[26]^ and hepatocellular carcinoma.^[27]^ miR-491-5p inhibits the proliferation and migration of A549 cells by forkhead box P4.^[28]^

The signaling pathways revealed by KEGG enrichment analysis and GO enrichment analysis from the 6 differentially expressed miRNAs in BALF group and 3 down-regulated differentially expressed miRNAs partly participate in inflammation and oxidative stress, which play a role in the pathogenesis of COPD. The results were consist with the studies of Renda T,^[29]^ Gaffey K,^[30]^ He S^[31]^ and Malhotra D.^[32]^ The other part is related to tumor, which is also reported.^[23–28]^ Further prediction analysis of target genes found that these differentially expressed miRNAs may be involved in the regulation of COPD by regulating mRNAs. These require further study to understand their roles in the pathogenesis of COPD. In addition, the roles of the 12 differentially expressed miRNAs both expressing in BALF and plasma instead in 2 control group, as well as the 159 differentially expressed miRNAs from comparing COPD group and BALF group required further exploration.

The deficiency of our study is that we did not further expand the population of differentially expressed differentially expressed miRNAs, nor did it directly measure the mRNA of COPD patients, and could not conduct miRNA-mRNA target gene pairing analysis. These will be further explored in subsequent studies.

## 5. Conclusions

Our findings demonstrate critical roles of the miRNAs in COPD molecular pathology. The data support a plausible mechanism that miRNAs may be involved in the development of COPD by affecting the inflammatory and oxidative stress. Moreover, hsa-miR-4748/CDC42/MAPK signaling pathway may contribute to the pathogenesis of COPD, providing a potential novel therapeutic strategy in COPD.

## Author contributions

**Conceptualization:** Qiaofa Lu.

**Data curation:** Weina Wang.

**Formal analysis:** Weina Wang, Lifen Du, Tian Qin.

**Funding acquisition:** Jianwu Hu.

**Investigation:** Qiaofa Lu.

**Methodology:** Jianwu Hu, Weina Wang, Tian Qin.

**Project administration:** Jianwu Hu.

**Resources:** Jianwu Hu, Lifen Du.

**Software:** Lifen Du, Tian Qin.

**Supervision:** Qiaofa Lu.

**Writing – original draft:** Jianwu Hu, Weina Wang, Lifen Du.

**Writing – review & editing:** Jianwu,Hu,Qiaofa Lu, Tian Qin.
